# Water droplet evaporation in varied gravity and electric fields

**DOI:** 10.1038/s41526-024-00396-2

**Published:** 2024-05-07

**Authors:** M. J. Gibbons, A. I. Garivalis, S. M. O’Shaughnessy, A. J. Robinson, P. Di Marco

**Affiliations:** 1 Department of Mechanical, Manufacturing & Biomedical Engineering, Trinity College Dublin, the University of Dublin, Dublin 2, Ireland; 2 DESTEC, University of Pisa, Largo Lazzarino 1, Pisa, 56122 Italy

**Keywords:** Mechanical engineering, Thermodynamics

## Abstract

Sessile water droplet evaporation in varied gravity and electric fields has been experimentally studied. Specifically, the influences of gravity and electric fields are investigated in the context of the heat flux distribution beneath the droplets, as well as the droplet mechanics and resulting shapes. Experimental testing was carried out during a European Space Agency (ESA) Parabolic Flight Campaign (PFC 66). The droplets tested evaporated with a pinned contact line, a single wettability condition, and varied droplet volume and substrate heat flux. The peak heat transfer was located at the contact line for all cases. The peak heat flux, average heat flux, and droplet evaporation rate were shown to vary strongly with gravity, with higher values noted for hypergravity conditions and lower values in microgravity conditions. The droplet thermal inertia was shown to play a significant role, with larger droplets taking more time to reach thermal equilibrium during the parabolic testing period. No significant impact of the electric field on the droplet evaporation was noted for these test conditions.

## Introduction

Phase change heat transfer in microgravity conditions is an active area of research, including flow boiling^1^ and pool boiling^2^, droplet evaporation^3–5^, combustion^6^, heat pump design^7^, propulsion^8^ and even solidification for 3D printing applications^9^. Interest is motivated by microgravity providing ideal conditions to investigate underlying physical phenomena^3,5,10–12^ and because there is a need to engineer more efficient, compact, and high-performance technologies for space applications^7,13,14^.

Droplet evaporation in terrestrial (normal) gravity conditions involves a complex interaction of diffusion within the substrate, buoyant convection in the gas and liquid phases, contact line evaporation, vapour diffusion, evaporative cooling at the liquid-gas interface, and possible Marangoni effects^11,15–24^. Regardless, the main driving force of the evaporation for a sessile droplet is the vapour concentration gradient across the droplet surface^3,11,25^. Furthermore, albeit a proportionately small region compared with the overall droplet size, the heat and mass transfer at the contact line plays an important role in droplet evaporation dynamics, and this has been shown conclusively in terrestrial gravity conditions^15,17–19,26^.

In microgravity conditions, with the absence of gravity-driven convection, the flow field within an evaporating droplet on a heated substrate is largely determined by Marangoni flow^25^. The main driving evaporation mechanism is the vapour diffusivity at the liquid-gas interface, and since mass diffusion is quite a slow process, this generally leads to lower evaporation rates^5^. Microgravity and its influence on contact line heat and mass transfer of evaporating sessile droplets is an underdeveloped topic.

Electrostatic forces^3,5,27–29^ have been investigated as a possible means of replacing the lost buoyancy force in microgravity. For a sessile droplet, the application of an electric field induces an electric stress at the liquid-vapour interface, deforming it and altering the contact angle^16^. In addition, electroconvection can be induced in the liquid and in the surrounding vapour atmosphere, resulting in a possible enhancement of the evaporation rate when gravity-driven convection is suppressed^3,5,27^. For normal gravity investigations^16^, the electrostatic force impacted the evaporation rate and the local heat flux distribution to the base of an evaporating droplet for both hydrophilic and superhydrophobic droplets when the electrostatic force acted to move the contact line. If the contact line remained pinned, no significant change in the local heat flux distribution was observed.

Possible influences of electric fields on HFE-7100 droplet evaporation in microgravity have only recently been reported^3,30^. Garivalis et al.^3^ and Dehaeck et al.^30^ showed in their sounding rocket campaign that the vapour concentration distribution is influenced by the electric field, and the electroconvection within the air-vapour phase enhanced the evaporation rate by about 25% for the observed droplets^3,30^.

While some progress has been made towards a better understanding of sessile droplet evaporation in microgravity, both with and without electric fields^3,5,27^, significant research is still required to understand the phenomenon fully. To this end, this study investigates the local heat transfer beneath an evaporating water droplet with and without electric fields in varied gravitational field strengths, including microgravity. Simultaneously, the droplet interfaces are experimentally and theoretically analysed to investigate the impact of gravity and the electric field on geometric properties and their subsequent influence on heat transfer and droplet mechanics. The results of the ESA Parabolic Flight Campaign (PFC 66) will be summarised, discussed, and compared. To the authors’ knowledge, for the first time, the local heat flux distribution underneath an evaporating droplet is measured in microgravity conditions.

## Results

### Experimental conditions and setup

**Fig. 1:**
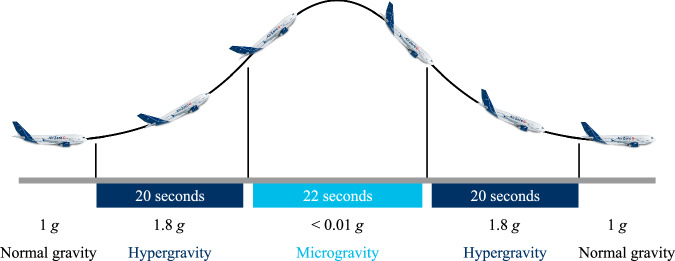
The different gravity phases during a parabola.

A single parabola, shown in Fig. 1, is ~62 s long, consisting of two 20 s phases of hypergravity (1.8${g}_{{\rm{z}}}$ or 17.65 m s^−2^) and a middle 22 s phase of microgravity (0.1 m s^−2^). During the microgravity phase, the gravity varies between ~±0.1 m s^−2^ due to small perturbations during the parabolic flight path.

**Fig. 2:**
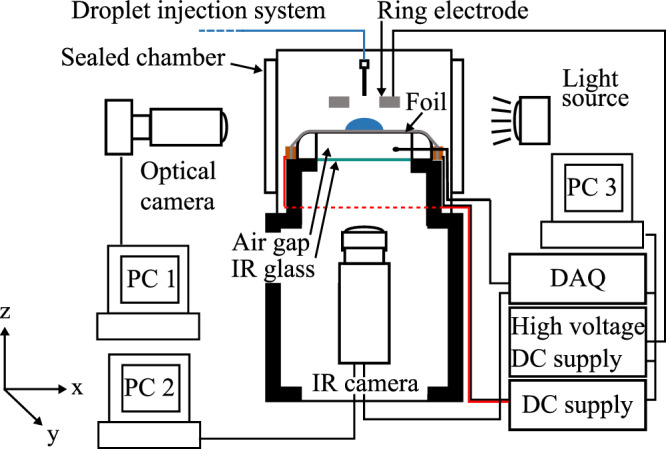
Schematic of the experimental apparatus.

The parabolic flight testing facility is shown in Fig. 2. A water droplet of controlled volume (3–59 µL) is deposited on the substrate before the initial hypergravity phase. The same drop is observed across successive parabolas as the drop evaporates slowly. To remove a drop and reset the evaporation process, the drop is vented outside the vessel by an evacuation system. During testing, the environmental conditions were 17 °C, 0.85 bar and <10% RH. The substrate is a 25-μm thick 316 stainless-steel foil (Goodfellow, 71 ${\rm{mm}}\times$ 35 ${\rm{mm}}\times$ 0.025 mm, P/N: 505-400-04) that is uniformly Joule heated. Two substrate-generated heat flux (${q}_{{\rm{gen}}}^{{\text{'}\text{'}}}$) magnitudes are explored 590 W m^−2^ and 890 W m^−2^.

### Droplet evaporation in varied gravity conditions

**Fig. 3:**
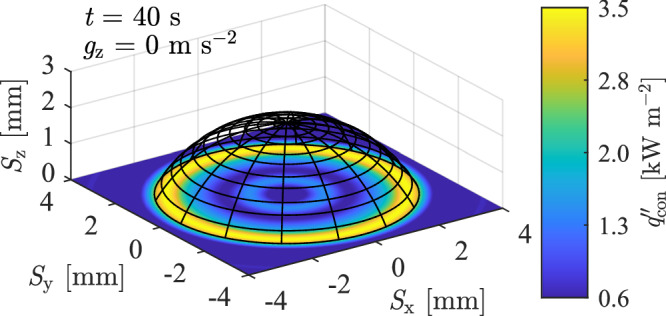
40 μL droplet 3D shape and heat flux distribution in microgravity on a heated foil (*q*″_gen_ = 590 Wm^−2^).

**Fig. 4:**
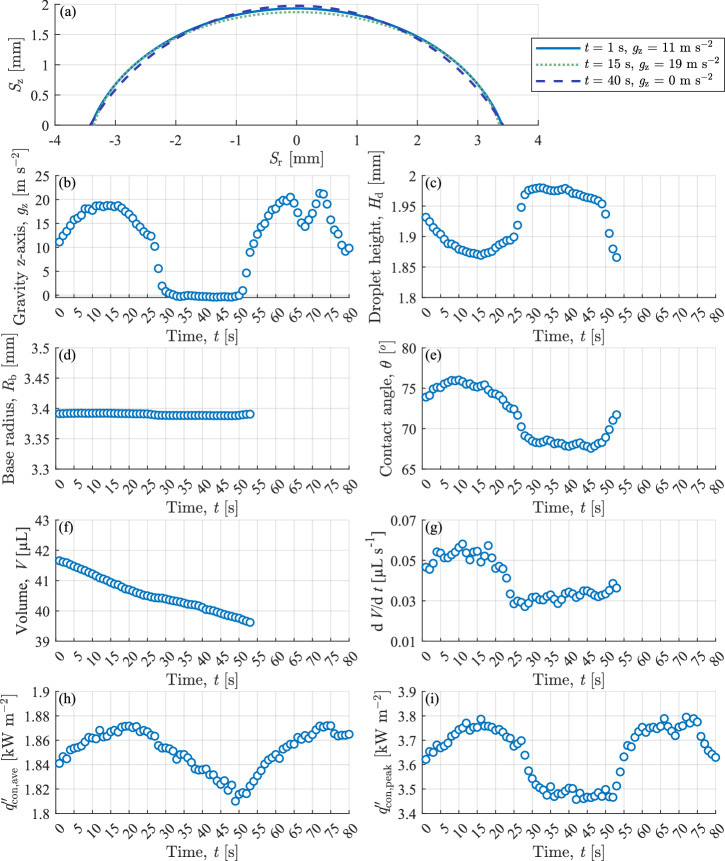
Droplet geometric and thermal characteristics in varied gravity with *q*″_gen_ *=* 590 Wm^−2^. The droplet base area was constant at ~38 mm^2^ during testing. **a** droplet liquid-gas interface in normal ($t$ = 1 s), hyper- ($t$ = 15 s), and microgravity ($t$ = 40 s) conditions. **b** vertical gravitational acceleration magnitude, **c** height, **d** base radius, **e** contact angle, **f** volume, **g** droplet evaporation rate, **h** average heat flux of wetted area and **i** peak heat flux.

Figures 3 and 4 show the geometric and thermal properties of an evaporating ~40 μL droplet on a surface with ${q}_{{\rm{gen}}}^{{\text{'}\text{'}}}=\,$ 590 W m^−2^ for a single parabola. Figure 4a, b show the three-dimensional droplet liquid-gas interface and heat flux distribution at time, $t$ = 40 s, during microgravity (0.1 m s^−2^) conditions. The peak heat flux (${q}_{{\rm{con}},{\rm{peak}}}^{{\text{'}\text{'}}}$) is noted at the droplet contact line for the microgravity condition and is consistent for all data. This agrees with previous ground-based research in the literature^16^.

Figure 4a shows the impact of the varied gravitational force on the droplet liquid-gas interface. A qualitative change in the droplet height and contact angle is observed. These geometric trends are further explored in Fig. 4c–f. Figure 4b plots the gravitational acceleration in the vertical z-direction ($${g}_{z})$$ that the droplet experiences through time. The droplet experiences hypergravity from $$t$$ = 5 s–25 s as the aircraft elevates (see Fig. 1) in preparation for the microgravity phase. From $$t$$ = 30 s–50 s, the droplet is exposed to microgravity ($$g$$ = 0 m s^−2^–0.1 m s^−2^) as the aircraft enters its free fall parabolic trajectory. Finally, from $$t$$ = 55 s–80 s, as the aircraft pulls out of the parabolic arc into level flight, the droplet experiences a second hypergravity phase.

Figure 4c–g explores the geometric properties of the evaporating droplet for varied vertical gravitational force. Optical data was gathered only for the initial hypergravity and microgravity phases ($t$ = 1 s–54 s). Figure 4d shows a near-constant base radius (${R}_{{\rm{b}}}$) over this interval, typical for hydrophilic droplet evaporation^15^. The opposite trends are noted for the droplet height $({H}_{{\rm{d}}})$ (Fig. 4c) and contact angle $(\theta )$ (Fig. 4e), with an increase in droplet height and a decrease in droplet contact angle as the gravitational acceleration decreases from 19 m s^−2^ to ~0 m s^−2^. This is due to the pinned contact line over this period; as the vertical gravitational force decreases, the surface tension forces minimise the droplet surface area, causing it to become a spherical cap, increasing the droplet height for a given volume. However, as the contact line is pinned, the contact angle decreases.

Two distinct droplet evaporation rates can be observed in Fig. 4f, g for the droplet volume $(V)$ over time. The inflexion in the curve divides the hypergravity (>17.65 m s^−2^) and microgravity (<0.1 m s^−2^) phases. The averaged evaporation rates for hyper- and microgravity are 0.054 µL s^−1^ and 0.032 µL s^−1,^ respectively, representing a decrease of 40% in the evaporation rate. This is similar to the bulk sessile droplet evaporation analysis observed by Garivalis et al.^3^ and Kumar et al.^5^. The local heat flux measurements further explain this change in evaporation rate over the parabola shown in Fig. 4h, i. The average heat flux (${q}_{{\rm{con}},{\rm{ave}}}^{{\text{'}\text{'}}}$) (Fig. 4h) is the average heat flux for the liquid-solid interface of the evaporating droplet. It is calculated by summing the individual heat flux for each pixel of the IR image and dividing by the wetted area.

The average (Fig. 4h) and peak (Fig. 4i) heat flux are observed to decrease in microgravity conditions (*t* = 30 s–50 s), resulting in a decrease in the thermal power transferred across the solid-liquid boundary of the droplet base. This decrease in thermal power results in the decrease in droplet evaporation rate noted in Fig. 4g.

The reduced evaporation rate in microgravity conditions observed here is in agreement with previous research^5^, and may be attributed to the reduced evacuation of the generated vapour from the droplet liquid-air interface^3^ and suppression of the liquid convective motion inside the drop if present. The air-side represents the dominant resistance between the source and sink. Heat and mass transfer from the sessile droplet surface occurs by a mix of diffusion and natural convection, with the latter exacerbated in hypergravity and suppressed in microgravity. As gravity approaches zero, the natural convection transport mechanism is eliminated, increasing the air-side thermal resistance whilst eliminating the air advection mechanism for transporting vapour from the interfacial region into the bulk. The net result is a significant increase in the overall resistance to heat and mass transfer and an associated decrease in the heat transfer to the base of the droplet from the heated surface.

Interestingly, the peak heat transfer, which occurs at the contact line as shown in Fig. 4i, trends closely with the change in the gravitational field (Fig. 4b) during the parabola, achieving quasi-steady state maximums and minimums for hyper- and microgravity conditions, respectively. In contrast, the average heat flux (Fig. 4h) to the droplet base does not achieve a steady state during the relatively short microgravity phase (t = 30 s–50 s). This can be attributed to the thermal inertia of the droplet, which is discussed in the next section.

## Discussion

The influence of the droplet’s thermal inertia can be approximated by the characteristic time, ${\tau}$, expressed in Eq. 1, which estimates the droplet’s responsiveness to a change in its thermal environment.

1 $$\tau =\frac{{m}{C}_{{\rm{p}}}}{{h}{A}_{{\rm{d}}}}$$

2 $$h=\frac{{q}_{{\rm{con}},{\rm{ave}}}^{{\rm{\text{'}\text{'}}}}}{{T}_{{\rm{d}}}-{T}_{{{\infty }}}}$$

In Eq. 1, $m$ is the mass of the droplet, ${C}_{{\rm{p}}}$ is the specific heat capacity of the liquid and ${A}_{{\rm{d}}}$ is the droplet base area. Here, $h$ is the characteristic convective heat transfer coefficient, denoted by Eq. 2, where ${T}_{{\rm{d}}}$ is the average temperature of the droplet base and ${T}_{\infty }$ is the ambient temperature of the test cell. This approach assumes that the droplet base temperature is characteristic of the entire droplet, which is sufficient for the present analysis given that it is well-documented that thermal resistance across the liquid-gas interface is much greater than the resistance associated with the liquid phase^31^.

**Fig. 5:**
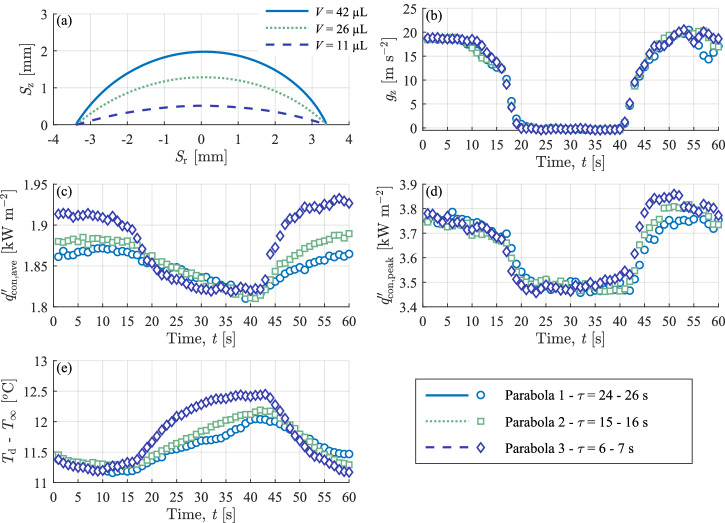
Droplet thermal inertia influence on dynamic heat transfer properties in a changing gravitational field with *q*″_gen_ = 590 W m^−2^. **a** Liquid-gas interface, **b** magnitude of the gravitational acceleration, **c** average heat flux, **d** peak heat flux and **e** ambient adjusted base droplet temperature.

Figure 5 investigates the impact of thermal inertia on droplet heat transfer dynamics in a changing gravitational field. Three droplets of different volumes, with constant and equal contact line radius and wall heat flux (${q}_{{\rm{gen}}}^{{\text{'}\text{'}}}=590\,{\rm{W}}\,{{\rm{m}}}^{-2})$, are contrasted. The initial volume of the three droplets are 42, 26 and 11 µL. Figure 5a depicts the liquid-gas interface of the three droplets at t = 30 s (middle of microgravity phase). Figure 5b plots the vertical gravitational field strength experienced by the respective droplets during their parabolas. The three parabolas have a close agreement in the magnitude and duration of the hypergravity and microgravity phases.

The associated characteristic time of these droplets calculated from Eqs. 1 and 2 are $\tau$ = 26 s, $\tau$ = 16 s and $\tau$ = 7 s, for the 42, 26 and 11 µL droplets, respectively. Albeit an approximation, it is clear that increasing the droplet volume, and thus thermal mass, increases the time required to respond to changes in its thermal environment. Notably, for the present study, this thermal response time can be of the same magnitude as the microgravity phase for larger droplets. One would thus expect the smallest droplet to approach a steady-state condition during the ~22 s microgravity interval, whereas the largest droplet should remain in a transient phase. Figure 5c shows the average convective heat flux into the droplet base regions, while Fig. 5e plots the average droplet base temperature difference with ambient for the three droplets. A moderately higher average heat flux (Fig. 5c) is noted for the smallest volume in the initial hypergravity phase (t = 1 s–17 s). This is consistent with the observations of Gibbons et al.^15^, where the thermal power to a hydrophilic droplet during the initial constant contact radius interval of evaporation was observed to increase despite its liquid-gas surface area decreasing. It is hypothesised that this can be attributed to a higher liquid-gas interface temperature for the thinner, lower volume droplet, resulting in a larger gradient with the ambient and commensurately higher heat transfer compared with bigger droplets of larger exposed surface area.

During the microgravity phase ($t$ = 20 s–40 s), the average heat flux to each droplet decreases. Comparing the three droplets, the smallest volume shows a rapid drop in average heat flux (Fig. 5c), dipping below that of the larger droplets, with a similarly rapid increase in ambient adjusted droplet base temperature (Fig. 5e), which rises notably above those of the larger droplets. This faster response of the smallest volume droplet (11 µL) is in line with it’s lower characteristic time, $\tau$ = 7 s calculated from Eq. 1.

The escalating base temperature and decreasing heat flux indicate an increased source-to-sink thermal resistance. For a constant wall heat flux boundary condition, an increasing thermal resistance causes the temperature to rise. For a constant wall temperature boundary condition, an increasing thermal resistance decreases the heat flux. In reality, between the constant wall heat flux and constant wall temperature boundary conditions, an increase in thermal resistance causes both the temperature to increase and the heat flux to decrease. Furthermore, both the average heat flux and ambient adjusted droplet base temperature achieve a steady state within the interval of microgravity conditions ($t$ = 32 s–42 s), which is anticipated for the 11 μL droplet due to its low characteristic time constant compared with the microgravity time interval. In contrast, the larger and higher thermal mass droplets, with time constants close to or exceeding the microgravity interval, are still transient by the end of microgravity conditions. However, they appear to be trending towards a similar heat flux and ambient adjusted droplet base temperature as the 11 $\,{\rm{\mu }}$L droplet. Regardless, it is clear that the absence of gravity reduces the effectiveness of the droplets in transporting thermal energy to the ambient surroundings.

A similar peak heat flux (Fig. 5d) is noted in hypergravity for all three droplets. This is sensible as the microscale contact line thickness and length should be similar for all three droplets, irrespective of the macroscale droplet geometries. In contrast to the average heat flux, as the droplets enter the microgravity phase ($t$ = 20 s–40 s), the peak heat fluxes of all droplets show close agreement with a transient response that closely tracks the gravity-time curves. As such, each reaches minimums of similar magnitude for the full duration of microgravity. Since the peak heat flux occurs in the micro-region between the bulk droplet and the absorbed film, it is not surprising that it is considerably more responsive than the bulk droplets, to the extent that it tends to change approximately in-phase with gravity.

**Fig. 6:**
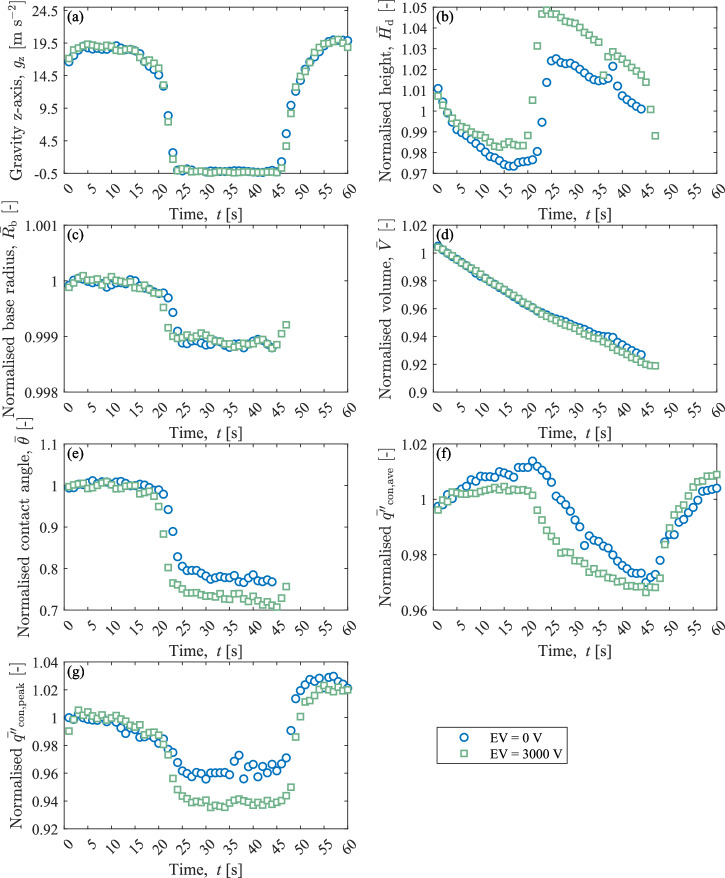
Electric field comparison for similar droplets for varied gravity conditions with *q*″_gen_ = 890 W m^−2^. Normalised values are with respect to Table 1. **a** Vertical gravitational acceleration magnitude, **b** normalised height, **c** normalised base radius, **d** normalised volume, **e** normalised contact angle, **f** normalised average heat flux and **g** normalised peak heat flux.

Figure 6 compares two droplets of similar volumes, geometric properties, and generated foil heat flux (${q}_{{\rm{gen}}}^{{\prime\prime}}=890\,{\rm{W}}\,{{\rm{m}}}^{-2}$) for two different electric field conditions of 0 and 3000 V throughout their respective parabolas. During testing, the external electric field is applied before the data acquisition and the parabolas start. Geometric and thermal values have been normalised by their initial values for comparison. Table 1 shows the initial droplet parameter values at the start of the hypergravity phase.

**Table 1:** Droplet comparison of initial values

	0 V	3000 V
Volume, $V$ [µL]	37	38
Height, ${H}_{{\rm{d}}}$ [mm]	1.53	1.57
Base radius, ${R}_{b}$ [mm]	3.69	3.73
Contact angle, $\theta$ [°]	56	58
Gravitational acceleration, $g$_z_ $[{\rm{m}}\,{{\rm{s}}}^{-2}]$	18.1	18.5
${q{^{\prime\prime}}}_{{\rm{con}},{\rm{ave}}}\,[\rm{kW}\,{m}^{-2}]$	2.71	2.80
${q{^{\prime\prime}}}_{{\rm{con}},{\rm{peak}}}\space[\rm{kW}\,{m}^{-2}]$	5.47	6.02

The z-direction gravitational acceleration (Fig. 6a) tracks closely for both cases. The normalised change in the droplet base radius (Fig. 6c) and volume (Fig. 6d) for varied gravity are similar with and without an applied electric field. However, a larger relative increase in the normalised droplet height (Fig. 6b) and a decrease in the normalised contact angle (Fig. 6e) are observed during the microgravity phase. As the contact line remains relatively pinned in both cases, the decrease in gravity allows the electric field to have a larger impact on the droplet liquid-gas interface. The electric field acts to reduce the droplet contact angle, increase its height, and elongate the droplet vertically.

**Fig. 7:**
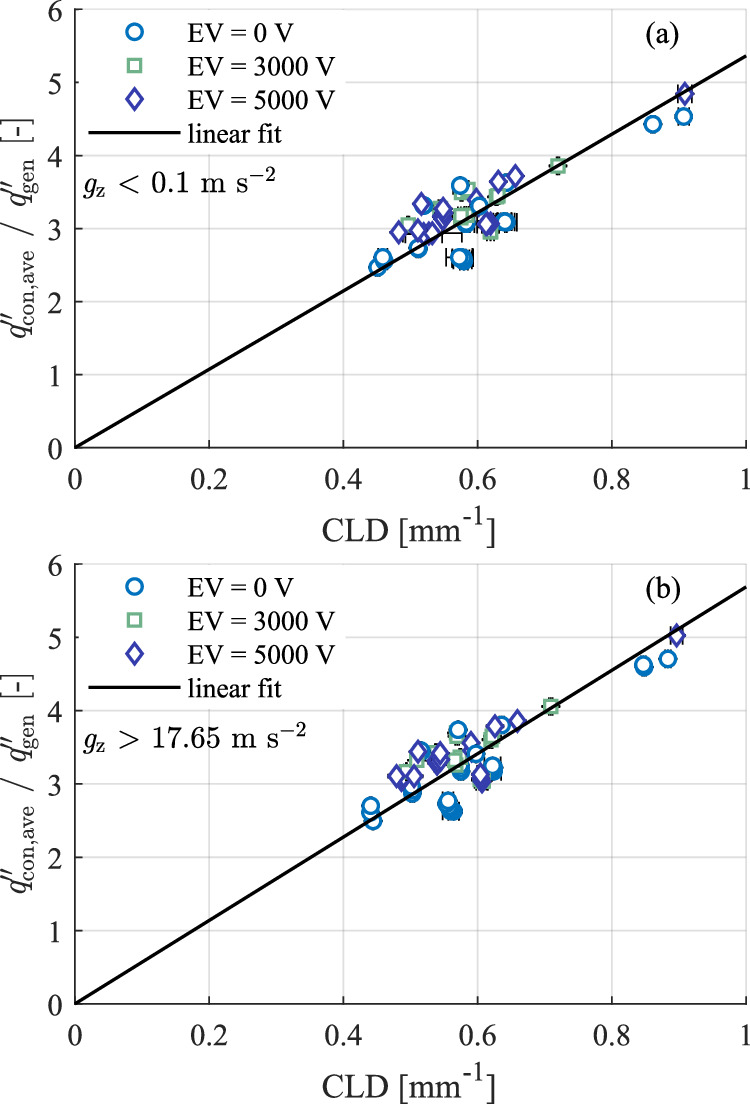
Contact line density vs. normalised average heat flux to the droplet base across solid-liquid interface for varied gravity and electrode voltage. **a** Microgravity and **b** hypergravity.

This result is similar to previous ground-based droplet evaporation studies^16,24^. No significant impact in the normalised average and peak heat flux of either droplet is observed, with any measured/calculated differences within the experimental uncertainty. Similar to Fig. 4, both the normalised average and peak heat flux closely track the gravitational field. The interaction of the electric field and heat transfer across the droplet solid-liquid interface is further investigated in Fig. 7.

As previously shown for droplet evaporation in normal gravity conditions in and out of an electric field^15,16^, the contact line density (CLD) is an important parameter in droplet evaporation. The contact line density (CLD)^18,32^ quantifies, in geometric terms, this relative proportion of the contact line to the overall base heat transfer region and is defined as:

3 $${\rm{CLD}}=\frac{{P}_{{\rm{CL}}}}{{A}_{{\rm{sl}}}}$$

where *P*_CL_ is the perimeter of the triple line, and *A*_sl_ is the base area of the droplet. When the base radius is large, the influence of the contact line heat transfer peak is confined to the region at the periphery of the droplet. However, when the base radius is small, the high heat transfer of the contact line dominates the heat transfer over the whole heat transfer area. Since the droplets are axisymmetric, ${P}_{{\rm{CL}}}={\rm{\pi }}{D}_{{\rm{b}}}$ and ${A}_{{\rm{sl}}}=0.25\cdot {\rm{\pi }}{{D}_{{\rm{b}}}}^{2}$, it follows that ${\rm{CLD}}\propto 1/{D}_{{\rm{b}}}$. Therefore, contact line density and reciprocal base diameter are equivalent for the studied droplets, but the CLD concept can be extended to non-axisymmetric cases, as verified by Horacek et al.^32^.

Figure 7 compares the CLD of the evaporating droplets captured during the parabolic flight campaign with the average droplet heat flux normalised by the generated foil heat flux. Micro- and hypergravity conditions are shown in Fig. 7a, b, respectively. All droplets maintained a fixed contact line over their respective parabolas. The final five seconds of the initial hypergravity ($t$ = ~5 s–10 s) and microgravity phases (*t* = ~35 s–40 s) are averaged in calculating the normalised heat flux (see Fig. 5b for reference).

Similar to previous normal gravity conditions^15,16^, the normalised heat flux for all applied electric fields collapses onto a common straight line. This indicates the overall thermal resistance is strongly related to the droplet base size in both hyper and microgravity conditions. Comparing gravitational field cases, a 6% increase in the normalised heat flux across the solid-liquid interface is noted for the hypergravity case.

No significant impact of the applied electric field on the normalised heat flux is observed. This is a similar result to previous terrestrial work by the present authors^16^, where no significant impact on the local heat flux to an evaporating droplet in an electric field was noted when the contact line remains fixed. However, recent research^3,30^ in the absence of gravity has shown the application of an electric field may induce some convection and influence evaporation. Garivalis et al.^3^ and Dehaeck et al.^30^ showed that an external electrostatic field in microgravity conditions alters the vapour cloud surrounding the evaporating droplet to a similar shape as that observed in normal gravity conditions. Kumar et al.^5^ also noted an increase in the average evaporation rate under microgravity conditions with an electric field. However, both cases only showed limited results owing to the challenges of acquiring large data sets for microgravity conditions from their experimental designs. Notably, both Garivalis et al.^3^ and Kumar et al.^5^ conducted their studies using sounding rockets, allowing much longer microgravity periods (~6.4 min). The use of a sounding rocket also enables the evaporation of a single drop without gravity level changes and, above all, a lower g-jitter (i.e., the gravity perturbation level). Moreover, they used constant wall temperature boundary conditions and a more volatile working fluid with heavier vapour (HFE-7100). All these differences may explain why a significant impact of the electric field is not observed here.

Sessile droplet evaporation in varied gravity and electric fields has been studied using thermal imaging and droplet shape analysis. The peak heat transfer was located at the contact line for all gravity and electric field cases. The peak heat flux at the contact line, the average heat flux and the droplet evaporation rate were shown to vary strongly with gravity, with higher values noted for hypergravity conditions and lower values in microgravity conditions. A 40% decrease in droplet evaporation rate was noted between hyper- and microgravity conditions. The reduced evaporation rate in microgravity conditions may be due to the suppression of convective motion in the drop and reduced evacuation of the generated vapour from the droplet liquid-air interface. Gravity is the driving force behind buoyant natural convection. As gravity is reduced, the vapour is transported away from the liquid-gas interface through diffusion alone, significantly increasing the droplet thermal resistance and decreasing the heat transfer to the base of the droplet from the heated foil.

The droplet thermal inertia was shown to play a significant role, with larger droplets taking a longer time to reach bulk thermal equilibrium than their smaller counterparts during the limited parabolic testing period. The peak heat flux at the contact line showed a transient response that closely tracks the gravity-time curves for all droplet volumes. No significant impact of the electric field on the droplet evaporation process was noted in the considered test conditions, but this may be due to the relatively short microgravity testing period (~22 s) in tandem with the strong role that the droplet thermal inertia plays. Further testing in extended microgravity conditions is required to understand better the impact of the electric field in microgravity conditions. Nevertheless, parabolic flights showed their limits for studying the relatively slow phenomena as drop evaporation. Other microgravity facilities, such as sounding rockets and the International Space Station (ISS), offer more stable microgravity levels, more controlled conditions, and longer experimentation periods. A drop evaporation experiment using water and HFE to be flown on board ISS is in preparation.

## Methods

### Hardware description

The stainless-steel foil substrate is uncoated, resulting in a hydrophilic wetting condition with water (advancing contact angle ${\theta }_{{\rm{A}}}$ = 85°, receding contact angle ${\theta }_{{\rm{R}}}$ = 55°). The underside of the foil is coated with a 10.5 μm thick layer of matte black paint to provide a known high emissivity surface for infra-red thermography measurements. The substrate properties are outlined in Table 2.

**Table 2:** Foil and paint properties

	Foil	Paint
Thickness, $\delta$ [µm]	25	10.5
Density, $\rho$ [kg m^-3^]	7960	1261
Thermal conductivity, $k$ [W m^−1^ K^−1^]	16.3	0.095
Specific heat, ${C}_{{\rm{p}}}$ [J kg^−1^ K^−1^]	502	2835
Roughness uncoated, ${\rm{Ra}}$ [nm]	60	-
Paint emissivity, ${\varepsilon }_{{\rm{p}}}\,$[-]	-	0.95

An infra-red (IR) transparent, anti-reflective coated, Germanium (Ge) window (40 ${\rm{mm}}\times$ 40 ${\rm{mm}}\times$ 2 mm) is located 15 mm below the stainless-steel foil, establishing an air cavity (30 ${\rm{mm}}\times$ 30 ${\rm{mm}}\times$ 15 mm) that acts as a thermal barrier. During testing, the stainless-steel substrate is directly heated by the Joule effect, establishing a uniform volumetric heat generation rate using a DC power supply connected to its ends. Two surface heat flux conditions were investigated: 590 and 890 W m^−2^. The temperature through the thickness of the foil and paint layers is assumed constant due to the low calculated Biot numbers $({\mathrm{Bi}}\ll \, 1)$^15,16,18^.

### Thermal and optical imaging systems

The droplet morphology is captured using an optical camera (Ximea, P/N: MQQ022MG-CM). The optical camera is mounted parallel to the heater surface with a pixel size of 4.7 μm. The temperature distribution of the heated substrate beneath the droplet is captured using a thermal imaging camera (FLIR, NETD: <30 mK, P/N: A655sc) fitted with a close-up lens (FLIR, P/N: T198059). The IR camera is mounted beneath the substrate, focused on the underside of the heated foil, and records at a resolution of 640 × 480 pixels, with a pixel size of 50 μm.

### Electric field

An electric field is established between the stainless-steel foil and a stainless-steel washer electrode placed directly above the droplet during experimentation, as depicted in Fig. 2, and connected to the positive side of a DC high-voltage power supply. The electrode is 1 mm thick, with inner and outer diameters of 4 and 10 mm, respectively. It is maintained at a height of 6 mm above the substrate. In this setup, the foil acts as a pseudo ground for establishing the electric field due to its low potential relative to the washer electrode voltage (EV). The washer electrode’s centre hole allows droplet deposition using an actuated needle and a syringe pump.

### Data acquisition and analysis

The substrate heat flux, optical camera, and thermal imaging camera are all automated using a custom-built LabVIEW programme. Thermal and optical data is acquired for 80 seconds from the start of each parabola at 50 Hz. The data reduction and analysis have been described in detail in previous work published by the present authors^15,16,18^. Therefore, only a brief description will be given here.

An element-wise energy balance is applied to the captured thermal image to calculate the heat flux distribution. Each element consists of a volume ${\rm{dx}}\times {\rm{dx}}\times \delta$, where ${\rm{dx}}$ is the pixel width of the IR camera, and $\delta$ is the thickness of the substrate. Uniform heat generation across the foil layer is assumed. A lumped capacitance analysis is performed as Bi $\ll$ 1 for both the foil and paint layers. Accounting for system losses, conjugate heat transfer (lateral conduction), and energy storage within the substrate yields:

4 $${q}_{{\rm{con}}}^{{\prime\prime}}={q}_{{\rm{gen}}}^{{\prime\prime}}-{q}_{{\rm{cond}}}^{{\prime\prime}}-{q}_{{\rm{rad}},{\rm{b}}}^{{\prime\prime}}+\left({k}_{{\rm{f}}}{\delta }_{{\rm{f}}}+{k}_{{\rm{p}}}{\delta }_{{\rm{p}}}\right)\left(\frac{{\partial }^{2}{T}_{{\rm{s}}}}{\partial {x}^{2}}+\frac{{\partial }^{2}{T}_{{\rm{s}}}}{\partial {y}^{2}}\right)-\left({\rho }_{{\rm{f}}}{C}_{{\rm{p}},{\rm{f}}}{\delta }_{{\rm{f}}}+{\rho }_{{\rm{p}}}{C}_{{\rm{p}},{\rm{p}}}{\delta }_{{\rm{p}}}\right)\frac{\partial {T}_{{\rm{s}}}}{\partial t}$$

where ${k}_{{\rm{f}}}$, ${k}_{{\rm{p}}}$, ${\delta }_{{\rm{f}}}$, ${\delta }_{{\rm{p}}}$, ${C}_{{\rm{p}},{\rm{f}}}$, and ${C}_{{\rm{p}},{\rm{p}}}$ are the foil and paint thermal conductivity, thickness, and specific heat capacity, respectively. The values for these parameters are given in Table 2. Equation 4 accounts for the generated flux within the metal substrate (${q}_{{\rm{gen}}}^{{\prime\prime}}$), the one-dimensional conduction (${q}_{{\rm{cond}}}^{{\prime\prime}}$) and the radiation (${q}_{{\rm{rad}},{\rm{b}}}^{{\prime\prime}}$) through the 15 mm air gap from the underside of the substrate. The final two terms in Eq. 4 are the heat transfer due to lateral conduction (${q}_{{\rm{lc}}}^{{\prime\prime}}$) and heat storage (${q}_{{\rm{cap}}}^{{\prime\prime}}$) within the substrate, respectively. ${q}_{{\rm{con}}}^{{\prime\prime}}$ is the heat flux transferred from the heated substrate into the base of the evaporating droplet. ${q}_{{\rm{con}}}^{{\prime\prime}}$ also encompasses the heat flux into the surrounding air in the far field (${S}_{{\rm{r}}}\,\gg \,{R}_{{\rm{b}}}$). The radial profile of the heat flux is determined by averaging lines taken radially from the centre of the droplet at 0.5° increments.

Data calculated from the droplet profile (height, radius, contact angle and volume) assume an axisymmetric droplet. A circularity (circularity = $(4{\rm{\pi }}{A}_{{\rm{sl}}})/({P}_{{\rm{CL}}}^{2})$) value of >0.8 is observed for optical data points, confirming the pseudo-axisymmetric assumption. Droplet circularity is calculated from the heat flux distribution data. The Bond number, ${\rm{Bo}},$ is defined by^33,34^:

5 $${\rm{Bo}}=\Delta \rho \,{g}_{{\rm{z}}}\,{R}_{0}/{\gamma }_{{\rm{l}}}$$

where $\Delta \rho$ is the density difference between the liquid and gas phases, ${R}_{0}$ is the characteristic radius, defined as the radius of a cylindrical drop of equal volume not in contact with the surface and ${\gamma }_{{\rm{l}}}$ is the droplet surface tension. A Bond number range of 0.01–1.55 is calculated from the data with no electric field. The largest Bond number is observed in the hypergravity phase, while the lowest Bond number corresponds to the microgravity conditions. In microgravity conditions, the measured volume from all optical data in non-electric field cases showed good agreement with the spherical cap model (${V}_{{\rm{sc}}}=\frac{1}{6}{\rm{\pi }}{H}_{{\rm{d}}}(3{R}_{{\rm{d}}}^{2}+{H}_{{\rm{d}}}^{2}$)) with an average difference of 1.1%. The electric Bond number, ${\rm{B}}{{\rm{o}}}_{{\rm{e}}},$ is defined by^35,36^:

6 $${\rm{B}}{{\rm{o}}}_{{\rm{e}}}={{\rm{\epsilon }}}_{0}\,{E}^{2}\,{R}_{0}/2{\gamma }_{{\rm{l}}}$$

where ${{\rm{\epsilon }}}_{0}$ is the vacuum permittivity, and $E$ is the average electric field between the electrodes (electrode voltage divided by electrode separation distance). An electric Bond number range of 0.01–0.11 is calculated from data tested with an electric field. The largest electric Bond number corresponded to an initial droplet volume of 59 μL and 5000 V applied voltage.

### Experimental uncertainty

The average experimental uncertainty is shown in Table 3. To calculate the average uncertainty, the individual uncertainty associated with each parabola is calculated and then averaged. All listed values are to a 95% confidence level^37^.

**Table 3:** Average expanded experimental uncertainty

Contact angle, $\theta$	± 40.0%
Volume, V	±8.9%
Height, $H$	±0.9%
Base radius, $R$_b_	±0.2%
Gravitational acceleration, $g$_z_	±0.1 ${\rm{m}}\,{{\rm{s}}}^{-2}$
${q}_{{\rm{con}}}^{{\prime\prime}}$	±16.8%

The standard uncertainty and degree of freedom of a single measurement is calculated from half the smallest measurement^37^, e.g., 0.5 pixels for optical data. The single measurement standard uncertainty and degree of freedom are applied to calculate the standard uncertainty for combined measurements such as droplet volume, contact angle, and heat flux. A correlation coefficient (${r}_{{\rm{cor}}}$) of one is assumed for time-averaged data to present the upper limit of the experimental uncertainty. Statistical and measurement experimental uncertainties are combined for the values given in Table 3^38^. The 40% contact angle measurement uncertainty stems from the interface perturbations during flight and uncertainty in calculating slopes between pixels with limited separation distance. The largest uncertainty in the heat flux energy balance is the lateral conduction term and stems from the variance in temperature measurement from adjacent pixels during data acquisition.

## Data Availability

The data collected during this study is available from the corresponding authors upon reasonable request.

## Code availability

The code used during this study is available from the corresponding authors upon reasonable request.
